# **Doctor Retention in Ireland - Where Are the Failings That Prolong the Problem?** Comment on "Doctor Retention: A Cross-sectional Study of How Ireland Has Been Losing the Battle"

**DOI:** 10.34172/ijhpm.2020.163

**Published:** 2020-09-01

**Authors:** Gozie Offiah, Frank Murray, Consilia Walsh

**Affiliations:** ^1^Royal College of Surgeons in Ireland, Dublin 2, Ireland.; ^2^National Doctors Training and Planning, Health Service Executive, Dublin 8, Ireland.

**Keywords:** Workforce, Doctor Retention, Migration, WHO Global Code, Ireland, Training

## Abstract

The issue of doctor retention has been a challenge in Ireland for many years. Poor working conditions including poor supervision, cost of training, bullying, worsening mentoring experiences and speciality specific issues are a substantial challenge faced by doctors in Ireland, thus leading to a higher degree of emigration. While some changes have been introduced to the system and have some positive effects, the root causes of doctor emigration have not been addressed. This commentary reviews the publication by Brugha et al published in the *IJHPM* in April 2020 on "Doctor Retention: A Cross-sectional Study of How Ireland Has Been Losing the Battle" and explains why the current system needs to change for the benefit of patient safety, doctor well-being and better patient care. Ireland’s Health Service Executive intends to take steps towards developing a new model of medical workforce to address the issue of recruitment and retention challenges within the healthcare system.

## Introduction

Following the publication of two reports by the Department of Health on the Strategic Review of Medical Training and Career Structures: Report on Medical Career Structures and Pathways Following Completion of Specialist Training and the National Doctor Retention Strategy report, several recent articles including the article by Brugha et al published in *IJHPM* have addressed the issue of doctor retention in Ireland.^1^ Brugha et al suggest that the national retention measures implemented in Ireland over the last few years have failed to effectively address stressful working conditions and unsatisfactory training for doctors, and thus requires a more diversified retention strategy.^2^ Their article raises important concerns regarding doctor retention in Ireland. This commentary outlines why the current system needs to change and explores two options: a more integrated medical workforce model and better working conditions to include a shorter duration of training in an aim to address doctor retention issues in Ireland.

## Medical Workforce Model for Ireland

In the last 20 years, there have been several reports published in relation to the medical workforce model for Ireland. In 2003, the Report of the National Task Force on Medical Staffing (Hanly Report) was published with recommendations for the Irish medical workforce.^3^ It recommended a consultant delivered service by a doubling of the number of consultants; a consultant to trainee ratio of approximately 2:1 and the need to assign consultants to a smaller number of hospitals, available for acute admissions on a 24/7 basis. It proposed that consultants would have substantial involvement in diagnosis, delivery of care and overall management of patients, allowing important clinical decisions to be made more rapidly and at a higher level. Thus, a model with an improved ratio of consultants to non-consultant hospital doctors (NCHDs) was suggested.^3^ In 2013, several reports including the report on Securing the Future of Smaller Hospitals: A Framework for Development was published and this referenced the need for better workforce planning.^4,5^ In 2014, a report by MacCraith, Strategic Review of Medical Training and Career structures referenced in the publication by Brugha et al highlighted the need to address recruitment and retention issues for NCHDs.^1^ This 2014 report was a seminal document for doctor retention in Ireland. The article in *IJPHM* by Brugha et al did not challenge the 2014 Strategic Review or the barriers after 5 years to implement the 25 recommendations from the report. In 2017, the high level Slaintecare report was published, advocating for the development of consultant delivered healthcare services to deliver care to the population of Ireland.^6^

All the above reports allude to a consultant delivered model of service which was outlined in the initial Report of the National Task Force on Medical Staffing over 17 years ago, yet many of the issues fundamental to this have not been addressed. Some indices have substantially deteriorated; for example the over-reliance of the Irish healthcare system on non-training NCHDs has deteriorated, combined with a lack of compliance with the World Health Organization (WHO) Global Code of Practice related to ethical recruitment in healthcare and doctor migration.^7^ Currently, in Ireland, approximately 50% of NCHDs are not on a training scheme, such that Ireland is the country with the highest proportion of non-training scheme doctors compared to like the United Kingdom, Canada, the United States, and Australia.^8,9^ Despite a huge reliance on these NCHD doctors, the system is struggling to retain these doctors, either due to challenges with poor career progression and working conditions. Thus many of the non-training scheme doctors are also leaving Ireland as they feel unfairly treated, thus creating a non-sustainable model for the delivery of healthcare.^10^

In addition to the challenges of a concrete model of medical workforce, several other factors have been identified to contribute to the retention issues of medical doctors to include working conditions, issues with bullying, inadequate resources and support, challenges with career progression including work life balance issues.^11,12^ In the study by Humphries et al, they identified a high level of migration to Australia since 2008 linked to the economic growth and better working conditions in Australia making it an attractive destination for Irish doctors.^11^

## Working Conditions in Ireland

In their research, Brugha et al identified that the highest negative ratings among doctors planning to leave permanently were stress levels in 62% of respondents, followed by poor staffing levels and high costs of training in 52% of respondents and lack of protected training in 48%.^2^ These challenges experienced by doctors working in the Irish system lead to poor working conditions and lack of time and opportunity for training.^13^ These issues not only lead to doctors leaving the health system but have patient safety implications. These doctors report that the Australian system works more efficiently with high staffing levels and thus making the job easier and less stressful.^11^ Recent studies report the poor working conditions for doctors in Ireland but more importantly the impact of these on doctors; with doctors feeling undervalued and underappreciated by policy makers.^13^

## European Working Time Directive

Another component that must be addressed is the issue of European Working Time Directive. A recent “Your Training Counts” report by the Medical Council of Ireland found that over a third of trainee respondents reported working 60 hours or more a week and thus were more likely to be involved in an adverse event.^14^ The same report also found that two-thirds of trainees who considered practicing abroad identified long working hours in Ireland as the main reason.^14^ Long working hours are an important doctor well-being issue as well as patient safety issue.

## Cost of Training and Duration of Training

Training cost was also identified in the study by Brugha et al as a challenge and a reason for migration. Trainees report the challenges with financial costs of living and training in Ireland.^9^ Consideration should be had on covering the full cost of training and working for doctors, similar in other healthcare systems like New Zealand.^15^

The duration of training in Ireland has been identified as significantly longer than other jurisdictions such as US and Canada. After graduation from medical school, doctors must complete a mandatory intern year, which enables them to apply for Basic Specialist Training, a 2 to 4-year programme. This is followed by competitive entry to a 3-6 year Higher Specialist Training (HST) programme, and successful completion of HST allows doctors to enrol on the specialist training register and compete for permanent consultant and general practitioner posts.^8^ This is a minimum of 7 years and a maximum of 11 years to consultancy, and a minimum of 5 years to complete General Practice training. This contrasts with the Canadian postgraduate medical training systems of two years for family medicine and four to five years for other specialities.^16^ Several Irish doctors find the Canadian type system much more attractive. Furthermore, the article by Brugha et al raises the issue of global healthcare worker crisis and its implications. The challenges of doctor retention is a global issue as already mentioned and is been addressed in many other jurisdictions in several ways such as the introduction of community-based learning and training in Thailand, monetary rewards in Cameroon and policy changes in many other countries.^17-19^

## Non-core Task Reallocation

 This refers to the re-allocating of basic non-medical tasks from NCHDs to other staff – specifically taking blood samples, inserting intravenous lines, performing electrocardiograms, discharging patients, and giving first doses of certain medication. Progress in reallocating these tasks has been painfully slow in Ireland, and is a consistent source of complaint for NCHDs, especially more junior NCHDs and needs to be addressed.

## Poor Supervision Experiences and Mentoring

Mentoring is a reciprocal relationship in which the mentor provides the mentee with guidance and support on professional and personal development to maximise their potential. There is generally an absence of formalised mentoring in Ireland for trainees. Several Irish reports have proposed that an integrated mentoring programme could help address gender diversity in medicine. The 2013 MacCraith report concluded from their review that mentoring is an important influence on personal development, career guidance, career choice and research productivity.^1,20^ While the availability of a formal mentoring programme for medical students could assist in selecting a career, it was noted that the lack of female surgeons to act as role models or mentors is a challenge that needs to be addressed first. The lack of adequate mentoring has also been reported as a barrier for career progression in surgery especially for female trainees.^21^ International research has also demonstrated the value of mentoring and yet several studies report the lack of mentoring programmes for medical students and medical doctors in most countries.^22^ Senior colleagues could play a significant role in increasing the retention of trainees and assisting them in navigating the career path to a permanent consultant appointment.

## Bullying

Bullying has been a challenge in Irish medical training as highlighted in a series of Your Training Counts reports by the Medical Council of Ireland. The same report in 2019 showed that 40% of respondents of a survey experienced some form of bullying, an increase in a similar report published four years earlier. Bullying perpetrators were most prominent among doctors at 58% while midwives and nurses represented 1/3 of perceived bullying perpetrators.^14^ The Irish Medical Council inspections of training sites also identified issues of bullying with junior trainees at internship level. The Irish Medical Council adopts a zero tolerance to bullying in the workplace and measures are being identified to tackle such behaviours. Bullying behaviour is one of the challenges faced by Irish medical trainees and contributes to several trainees leaving the workforce and migration abroad.

## Specialty Specific Issues

 Differences across the specialties were identified by Brugha et al in the factors and timing of migration of doctors and these warrants further investigation. Of note those planning careers in Anaesthesiology were most likely to leave and return, while those planning to leave permanently were psychiatry trainees who had completed HST.

## Conclusion

 Despite the many strategic reports produced in the last few years, there has been a lack of implementation of many of the recommendations highlighted to improve the medical workforce, and a widespread perception by NCHDs that the situation has not improved overall. A primary aim is to retain Irish trained graduates within Ireland and to maintain the WHO global code. An improvement on the ratio of trained to non-trained medical staff would decrease the reliance on junior doctors and non-training scheme doctors and lead to improved changes to work practices. The proposed model of consultant delivered care is consistent with the HSE Corporate Plan 2015-2017 goal to provide fair, equitable and timely access to quality, safe health services that people need.

 While the article by Brugha et al is a welcome reminder and an up to date analysis of junior doctor career intentions, it has not presented any new data that was not already in the Irish reports and thus a review of the challenges to implementation of the recommendations would be a welcome addition to the literature.

## Ethical issues

 Not applicable.

## Competing interests

 Authors declare that they have no competing interests.

## Authors’ contributions

 GO is the author of the paper. CW and FM undertook critical revision of the manuscript.

## Authors’ affiliations

^1^Royal College of Surgeons in Ireland, Dublin 2, Ireland. ^2^National Doctors Training and Planning, Health Service Executive, Dublin 8, Ireland.
